# Morphogenesis of *Strongyloides stercoralis* Infective Larvae Requires the DAF-16 Ortholog FKTF-1

**DOI:** 10.1371/journal.ppat.1000370

**Published:** 2009-04-10

**Authors:** Michelle L. Castelletto, Holman C. Massey, James B. Lok

**Affiliations:** Department of Pathobiology, School of Veterinary Medicine, University of Pennsylvania, Philadelphia, Pennsylvania, United States of America; NIAID/NIH, United States of America

## Abstract

Based on metabolic and morphological similarities between infective third-stage larvae of parasitic nematodes and dauer larvae of *Caenorhabditis elegans*, it is hypothesized that similar genetic mechanisms control the development of these forms. In the parasite *Strongyloides stercoralis*, FKTF-1 is an ortholog of DAF-16, a forkhead transcription factor that regulates dauer larval development in *C. elegans*. Using transgenesis, we investigated the role of FKTF-1 in *S. stercoralis'* infective larval development. In first-stage larvae, GFP-tagged recombinant FKTF-1b localizes to the pharynx and hypodermis, tissues remodeled in infective larvae. Activating and inactivating mutations at predicted AKT phosphorylation sites on FKTF-1b give constitutive cytoplasmic and nuclear localization of the protein, respectively, indicating that its post-translational regulation is similar to other FOXO-class transcription factors. Mutant constructs designed to interfere with endogenous FKTF-1b function altered the intestinal and pharyngeal development of the larvae and resulted in some transgenic larvae failing to arrest in the infective stage. Our findings indicate that FKTF-1b is required for proper morphogenesis of *S. stercoralis* infective larvae and support the overall hypothesis of similar regulation of dauer development in *C. elegans* and the formation of infective larvae in parasitic nematodes.

## Author Summary

Parasitic nematodes are an important threat to public health in much of the world. Understanding how these worms find and invade their hosts may lead to improved therapies. The infectious forms of many parasitic nematodes developmentally arrest as infective third-stage larvae that require hosts to reactivate. Development of these larvae has been compared to that of the diapausing dauer larvae of *Caenorhabditis elegans*. Our lab studies the development of the human nematode parasite *Strongyloides stercoralis*. We identified *S. stercoralis'* FKTF-1 as an ortholog of DAF-16, a forkhead transcription factor controlling dauer larval development in *C. elegans*. Transgenes were introduced into *S. stercoralis* to investigate the possibility that FKTF-1 regulates development of its infective larvae. We discovered that recombinant FKTF-1b tagged with GFP localizes to specific tissues remodeled in infective larvae. Furthermore, mutant forms of FKTF-1b designed to interfere with endogenous FKTF-1b function resulted in incomplete development of the infective larval structures and prevented some transgenic larvae from arresting in the infective stage. Indicating that FKTF-1b is required for the proper development of *Strongyloides stercoralis* infective larvae, our findings support the hypothesis of similar controls over parasitic and free-living nematode development and pave the way for further comparative studies.

## Introduction

Parasitism among nematodes appears to have arisen multiple times throughout evolution [1]. However, the exact mechanism by which nematodes developed parasitic life histories is unknown. Altering gene regulation through variation in conserved signaling systems, is a potential mechanism by which a free-living species might develop characteristics required for parasitism [2]. Insulin-like signaling regulates metabolism and lifespan in a variety of organisms including nematodes, insects and mammals [3],[4]. In *Caenorhabditis elegans*, this signaling pathway mediates entry into the dauer larvae diapause by negatively regulating DAF-16, a forkhead transcription factor type O (FOXO) [5]. Biological requirements of *C. elegans* dauer larvae include increased resistance to stress and a metabolism altered to allow the animal to persist, potentially for months, in unfavorable environments [6]. Infective larvae of parasitic nematodes, such as *S. stercoralis*, have similar requirements for survival prior to host finding. The ‘dauer hypothesis’ recognizes the common physiological characteristics of dauer larvae and parasitic infective larvae, and proposes that the same molecular genetic mechanisms control the morphogenesis of both forms [7].

The life cycles of *Strongyloides* and *Parastrongyloides* spp., unusual among the parasitic nematodes, alternate between free-living and parasitic generations [8],[9]. First-stage larval progeny of parasitic *S. stercoralis* females typically develop into free-living adults unless triggered by genetic, environmental or host-associated conditions to develop directly into infective third-stage larvae (L3i) [10]. Progeny of the free-living generation of *S. stercoralis* are uniformly fated to become L3i that invade the host and develop into parasitic females. Previous work identified the FOXO encoding gene *fktf-1 (forkhead transcription factor-1)* as the ortholog of *C. elegans daf-16* in *S. stercoralis* [11]. In heterologous rescue experiments, a transgene construct designed to express FKTF-1b (isoform b) partially restored DAF-16 function to *C. elegans daf-2;daf-16* double mutants [12] rescuing the dauer development phenotype. These data indicate that *fktf-1b* encodes a working forkhead transcription factor that can function in insulin-like signaling to regulate L3 development in *C. elegans*.

The more relevant question of whether FKTF-1b regulates infective larval development in *S. stercoralis* itself can now be addressed using new methods for transgenesis in this parasite [13],[14]. In the present study, we transformed free-living adult female *S. stercoralis* with constructs encoding a 2.6 kb *fktf-1β* promoter controlling expression of GFP::FKTF-1b fusion proteins. We then examined first-stage larval progeny of these female worms for anatomical and intra-cellular localization of GFP-linked proteins and for phenotypes associated with expression of transgenes encoding mutant forms of FKTF-1b.

## Results/Discussion

### Anatomical expression patterns of *fktf-1β*

**Figure 1:**
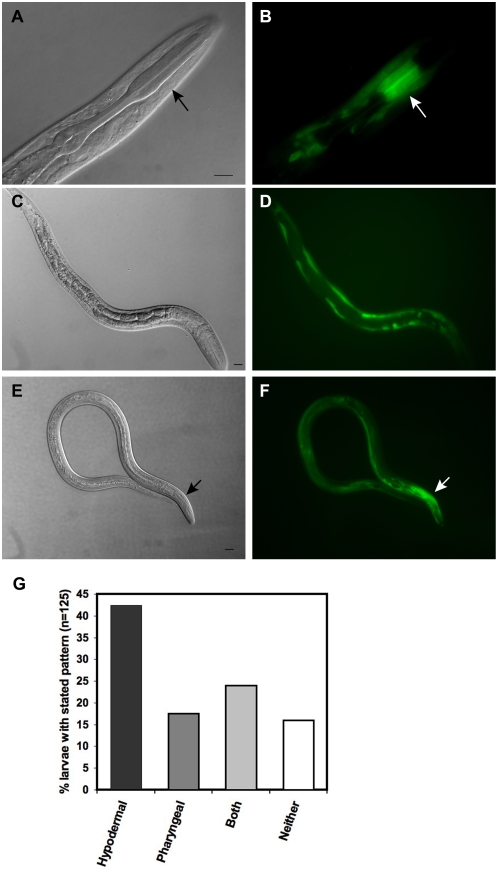
Anatomical expression patterns of *fktf-1β::gfp::fktf-1b*. DIC and fluorescence images of transgenic *S. stercoralis* larvae. Each DIC image is a separate individual. All scale bars = 10 µm. (A,B) Transgenic first-stage larvae with GFP expression in the procorpus (arrow) of the pharynx. (C,D) Expression of the GFP::FKTF-1b(wt) transgene in the hypodermis of an L1. (E,F) Transgenic L3i expressing the GFP::FKTF-1b(wt) fusion protein in the hypodermis and a narrow band in the pharynx (arrow). (G) Sites of *gfp* expression under the direction of the *fktf-1β* promoter in 125 transgenic first-stage larvae from five or more experiments expressing *gfp* reporters under the direction of *fktf-1β* promoter. Due to variations in transformation rates between experiments, all transgenic larvae were pooled to quantify expression patterns. χ^2^ test, P = 2.9E-14.

First, we asked whether the localization of *fktf-1β* expression in *S. stercoralis* mimics that of *C. elegans daf-16β*, which is expressed primarily in the pharynx and body neurons [5]. First-stage *S. stercoralis* larvae expressed *fktf-1β::gfp::fktf-1b* (Figure S1) predominantly in the procorpus of the pharynx (Figure 1A, and Figure 1B, arrow) and the hypodermis (Figure 1C and 1D). These expression patterns continued into the L3i (Figure 1E and 1F). In *S. stercoralis*, remodeling of the short, trilobed rhabditiform pharynx of the L1 into the long, cylindrical filariform pharynx of the L3i is a hallmark of the transition to infectivity [15]. The rhabditiform pharynx, found in all free-living stages, has three main components: the procorpus, the isthmus and the terminal bulb. The procorpus of the pharynx is the muscular region anterior to the narrow isthmus and is primarily responsible for food intake [15]. The pharynx of the non-feeding L3i, is not contractile and has no readily identifiable lobes [15]. Interestingly, expression of the *fktf-1β* reporter construct in the filariform pharynx was restricted to a band (Figure 1F arrow) analogous to the procorpus of a rhabditiform pharynx. The hypodermal cell layer is responsible for secretion of the cuticle in a stage specific manner [16]. The infective larval cuticle must not only protect the L3i, it must also allow the L3i to sense the presence of a host and secrete molecules facilitating invasion. Although the expression patterns of the fusion protein in L1 varied somewhat (Figure 1G), the fact that the predominant sites of expression were the pharyngeal procorpus and the hypodermis bolsters confidence that the endogenous *fktf-1β* promoter is active in these tissues in wild-type larvae. This pattern of expression is consistent with a role for FKTF-1b in the development of structures characteristic of infective larvae.

### FKTF-1b is regulated via phosphorylation

**Figure 2:**
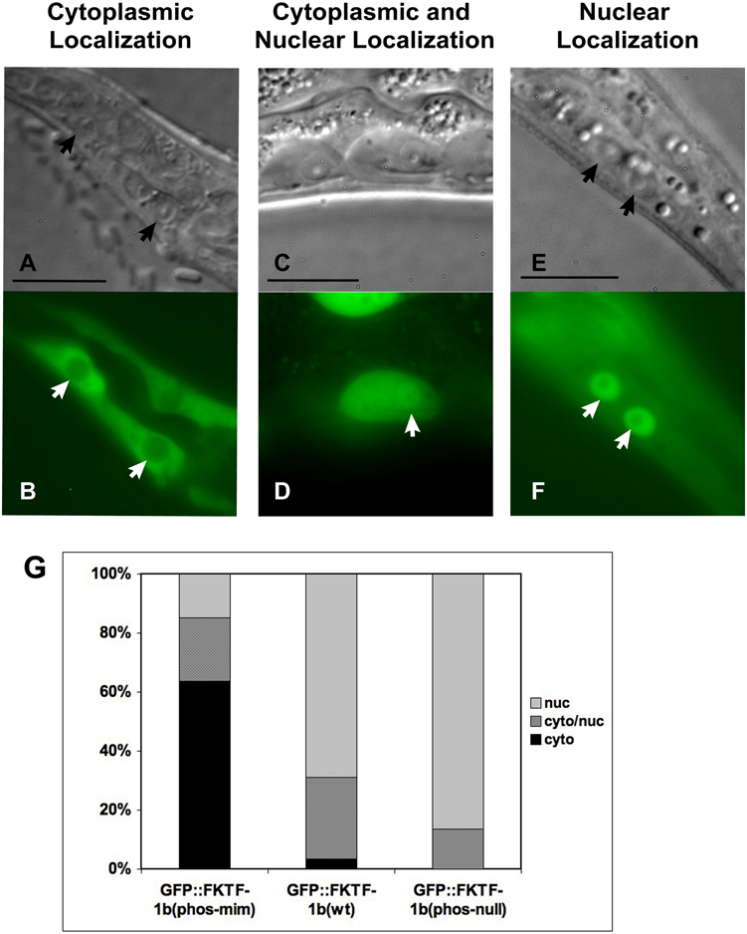
Intra-cellular localization of GFP::FKTF-1b phosphorylation mutants. DIC and fluorescence images of representative hypodermal cells of transgenic *S. stercoralis* L1s. In all images, the nucleus is identified with an arrow. All scale bars = 10 µm. (A,B) L1 expressing pPV244(S238E/T240E), the “phospho-mimetic” GFP::FKTF-1b with fluorescence in the cytoplasm. (C,D) L1 expressing pPV234, GFP::FKTF-1b(wt), with fluorescence in both the cytoplasm and the nucleus. (E,F) L1 with strong nuclear localization of the FKTF-1b(4A) fusion protein. (G) Percentages of hypodermal cells in transgenic larvae with intra-cellular localization of GFP classified as “cytoplasmic”, “cytoplasmic/nuclear”, or “nuclear”. Results include transgenic larvae from at least two separate microinjection experiments per transgene. Phospho-mimetic: n = 47 GFP+ cells in 11 larvae. GFP::FKTF-1b: n = 29 GFP+ cells in 8 larvae. Phospho-null: n = 37 GFP+ cells in 8 larvae. χ^2^ test P = 1.23E-10.

Insulin-like signaling negatively regulates the function of forkhead transcription factors, including DAF-16, via phosphorylation of serines or threonines at specific sites by Akt/PKB kinases [17]. To ascertain similar post-translational regulation of FKTF-1b, we transformed *S. stercoralis* with vectors encoding mutant versions of GFP::FKTF-1b that were predicted to behave as either constitutively phosphorylated or non-phosphorylated forms of the protein. Substitution of charged residues, either aspartic or glutamic acids, for serines at Akt/PKB phosphorylation sites in the forkhead domain of human FOXOs is sufficient for disruption of DNA binding by these proteins and for their export from the nucleus [18],[19]. Homologous ‘phospho-mimetic’ mutations in predicted Akt/PKB sites of FKTF-1b also resulted in constitutive export of the fusion protein GFP::FKTF-1b(S238E/T240E) (encoded by pPV244, Figure S1) from nuclei of hypodermal cells in transgenic *S. stercoralis* L1 (Figure 2A and 2B and 2G). Likewise, disruption of all four predicted Akt/PKB sites in FKTF-1b by substitution of the neutral amino acid alanine for critical serine or threonine residues (see pPV243, Figure S1) resulted in strongly enhanced nuclear localization of the ‘phospho-null’ fusion protein GFP::FKTF-1b(4A) (Figure 2E–2G). These data indicate that FKTF-1b's intra-cellular localization, and thereby its access to genomic response elements, is regulated by phosphorylation in a similar manner to DAF-16 and other FOXO-class transcription factors.

### Dominant interfering transgenes cause altered intestinal morphology in L1s

Its anatomical localization and intra-cellular trafficking support the hypothesis that FKTF-1b is an ortholog of DAF-16 and that through it, insulin-like signaling regulates *S. stercoralis'* larval development. More conclusive testing of this hypothesis requires experimental manipulation of gene function and evaluation of phenotypic outcomes. Thus far, *S. stercoralis*, like many other parasitic nematodes, has proven insensitive to targeted gene silencing via RNAi [20]. Therefore, we opted for an approach based on transgenesis in which we express altered forms of FKTF-1b designed to interfere with the function of the endogenous transcription factor. Two such mutant proteins, encoded by plasmids pPV251 and pPV298, respectively (Figure S1), are tagged with GFP and carry the four ‘phospho-null’ mutations described above, causing them to be sequestered in the nucleus where they presumably out-compete native FKTF-1b for response elements in the genome. In addition, both mutant proteins are truncated within the C-terminal domain immediately downstream of the fourth regulatory phosphorylation site, ablating key transactivator binding motifs. In one of the dominant interfering proteins, encoded by pPV251 and dubbed GFP::FKTF-1b(dominant-repressor), the truncated C-terminal domain is fused to the repressor domain of Ce-PIE-1, a protein responsible for the transcriptional repression characterizing the germline precursor of *C. elegans* [21]. In the other mutant protein, encoded by pPV298 and dubbed GFP::FKTF-1b(dominant-negative), the truncated C-terminal domain is not linked to Ce-PIE-1.

**Figure 3:**
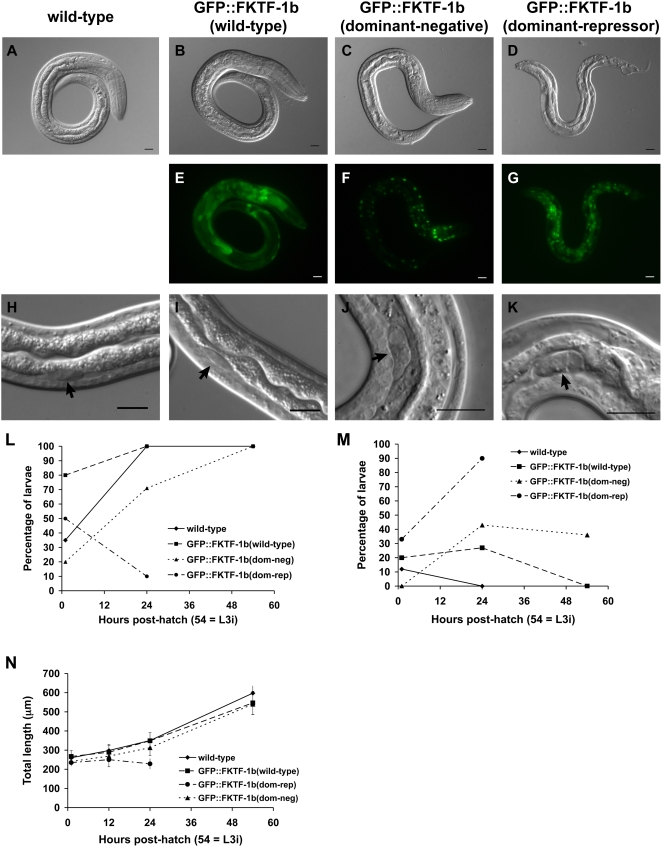
*S. stercoralis* transgenic L1s 24 hours post-hatch. Representative images of *S. stercoralis* wild-type and transgenic larvae at 24 hours post-hatch. Each DIC image is a separate individual. All scale bars = 10 µm. By 24 hours, the wild-type (A) and GFP::FKTF-1b(wt) (B) larvae have grown to comparable lengths with similar numbers of intestinal granules. (C) GFP::FKTF-1b(dom-neg)–expressing larvae are a similar length but show reduced intestinal granularity. (D) GFP::FKTF-1b(dom-rep)–expressing animals exhibit almost a complete loss of intestinal architecture and do not survive past 24 hours. Comparable levels of transgene expression were seen in the transgenic animals at 24 hours (E–G). (H–K) Higher magnification images of intestinal cells near primordial gonad (arrow) showing fewer storage granules in the GFP::FKTF-1b(dom-neg) (J) and the loss of cell integrity in GFP::FKTF-1b(dom-rep)– (K) expressing larvae at 24 hours. (L) The presence of storage granules in the intestinal cells of wild-type and transgenic larvae was scored+if the majority of cells near the primordial gonad (arrow) contained granules. (M) Wild-type and transgenic larvae were scored for overall intestinal defects including structural damage and abnormal morphology. Larvae expressing either of the dominant-interfering transgenes exhibited intestinal defects at all timepoints (see Table S2 for counts). (N) All larvae were measured from the tip of the buccal cavity to the tip of the tail using the ImageJ program [27]. A minimum of 10 larvae per category per timepoint, with the exception of GFP::FKTF-1b(wild-type) L3i where n = 8, were examined and measured (Table S2). At 1 hour post-hatch, larvae expressing either of the dominant-interfering transgenes were slightly shorter than the wild-type larvae (P<0.05). At 24 hours post-hatch, only the GFP::FKTF-1b(dominant-repressor) transgene–expressing larvae were significantly shorter than the wild-type (P<0.01). The transgenic L3i expressing the GFP::FKTF-1b(dom-neg) transgene were not significantly shorter than the wild-type L3is (P>0.05).

Upon hatching, larvae expressing either of the dominant interfering constructs were shorter but virtually identical in form to larvae expressing GFP-tagged wild-type FKTF-1b at similar levels (Figure S2), indicating that the *S. stercoralis* transcription factor, like DAF-16 in *C. elegans* [22], does not play a significant role in embryonic development. By contrast, at 24 hours, *S. stercoralis* L1 expressing either of the dominant interfering mutants of FKTF-1b exhibited phenotypic changes in the form and apparent function of their intestinal cells, with these being most evident in larvae expressing the dominant-repressor construct. These phenotypes ranged from flattening of the normally apically rounded intestinal cells and a decrease in the number of cytoplasmic storage granules in the presence of GFP::FKTF-1b(dominant-negative) (compare Figure 3J to Figure 3H and Figure 3I) to an almost complete loss of intestinal cell architecture and of cytoplasmic storage granules in the presence of the GFP::FKTF-1b(dominant-repressor). Perhaps due to compromised intestinal cell function, *S. stercoralis* L1 expressing GFP::FKTF-1b(dominant-repressor) exhibited significant (P<0.01) growth retardation at 24 hours (Figure 3N). Owing to the severity of the associated phenotypes, none of the larvae expressing GFP::FKTF-1b(dominant-repressor) survived beyond the L1. The fact that *S. stercoralis* L1 expressing comparable levels of wild-type FKTF-1 tagged with GFP (Figure 3B, 3E, and 3I) were morphologically similar to untransformed larvae (Figure 3A and 3H) argues against the observed phenotypes being due to non-specific effects of recombinant protein expression. Therefore, it is clear from these findings that FKTF-1 is necessary for normal development of intestinal cells in pre-infective larvae of *S. stercoralis* and specifically for accumulation of storage granules, which may contain reserves necessary for survival of the L3i.

### Dominant interfering transgenes cause aberrant morphogenesis in *S. stercoralis L3*

**Figure 4:**
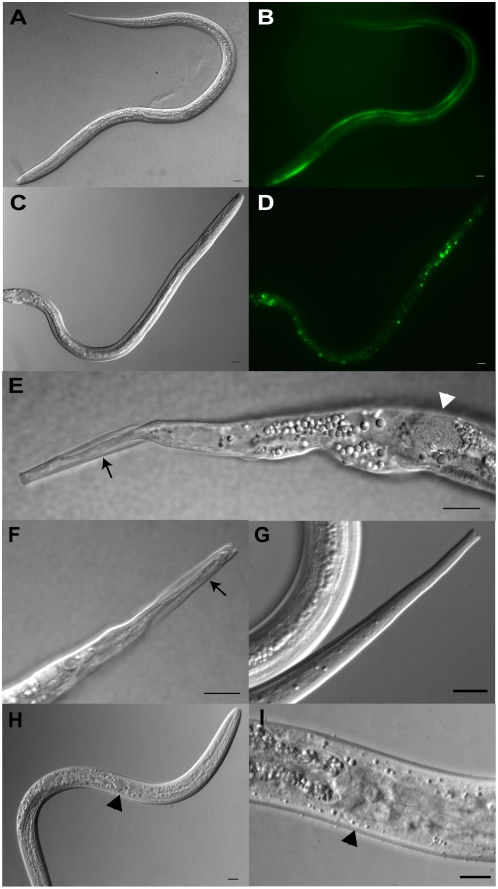
Third-stage *S. stercoralis* larvae expressing dominant interfering transgenes. DIC and fluorescence images of transgenic L3s [n = 11 for GFP::FKTF-1b(dominant-negative), n = 8 for GFP::FKTF-1b(wild-type)]. All scale bars = 10 µm. Except where noted, each DIC image is a separate individual. (A,B) An L3i expressing the GFP::FKTF-1b(wt) transgene. (C,D) An L3 expressing the GFP::FKTF-1b(dominant-negative) transgene. (E) Higher magnification image of the tail of the L3 in (C) showing an L4 tail inside the characteristic L3i cuticle (arrow). Bacterial mass in the posterior intestine (white triangle) indicating failure of intestine to become radially constricted and blocked. (F) A second transgenic L3 showing the free-living L4 tail inside the L3i cuticle. (G) Wild-type L3i tail for comparison to transgenic tails. (H) A third transgenic L3 expressing the dominant-negative transgene with an elongated rhabditiform pharynx, with an intestine that is neither radially constricted nor blocked. (I) Grinder-like structure (black triangle) at the pharynx-intestine junction of (H). This structure is absent in wild-type L3is.

With regard to the dauer hypothesis and the role of insulin signaling in infective larval development, the most significant results in the present study were the morphogenetic changes seen in L3 expressing the GFP::FKTF-1b(dominant-negative) transgene. Under the null hypothesis, all of our transgenic larvae should develop to L3i. While L3i expressing the wild-type GFP::FKTF-1b fusion protein were morphologically identical to their non-transgenic counterparts (Figure 4A and 4B, compare to Figure S3A), L3 expressing the dominant-negative transgene (Figure 4C and 4D) exhibited some indications of bypassing developmental arrest and failing to undergo the pharyngeal and intestinal remodeling characteristic of L3i. Three of the 11 transgenic L3 appeared to initiate an aberrant molt to the fourth stage as evidenced by the existence of a pointed tail inside a notched L3i cuticle cast (Figure 4E and 4F compared to wild-type Figure 4G). The notched tail is characteristic of infective larvae and is created by pairs of ‘L3i-specific’ alae [15]. Another L3 expressing the dominant-negative construct exhibited an elongated rhabditiform pharynx complete with a grinder-like structure (Figure 4H and 4I) instead of the expected filariform pharynx (Figure S3B and Figure S3C). Incomplete remodeling of the rhabditiform pharynx and initiation of a supernumerary molt in culture are consistent with expression of the interfering FKTF-1b transgenes in the pharyngeal procorpus and the hypodermis. Initiation of ecdysis by L3 in combination with retention of some rhabditiform pharyngeal characteristics as we observed suggests that worms expressing GFP::FKTF-1b(dominant-negative) were developing in the direction of a second-generation free-living L4. While such a form occurs in some strongyloidoid species (e.g. *Strongyloides planiceps*, *Parastrongyloides trichosuri*), it does not exist in the natural life cycle of *S. stercoralis* [8],[23].

Five of the 11 transgenic L3 expressing the dominant-negative construct exhibited changes consistent with a failure to remodel the free-living intestine into the darkened, radially constricted intestine of the L3i. In some cases, the L3 intestine retained bacteria (Figure 4E) and in others, it failed to constrict and close (Figure 4I to the left of the black triangle). The incompletely remodeled intestine seen in the transgenic L3 is consistent with the defects in intestinal structure seen in the L1. Together, these data indicate that FKTF-1b is required for the proper remodeling of the pharynx and the intestine of a free-living larva into structures characteristic of the infective larva.

Our findings support the ‘dauer hypothesis’ [7] by showing that the forkhead transcription factor FKTF-1b, presumably under the control of insulin-like signaling, regulates infective larval development in *Strongyloides stercoralis* in a manner similar to the dauer regulatory functions of DAF-16 in *Caenorhabditis elegans*. Furthermore, in this study, we have demonstrated the utility of transgenesis in *S. stercoralis* for investigation not only of temporal and spatial patterns of gene expression, but also of endogenous gene function. This work opens new avenues of inquiry into the genes involved in the shift between free-living and parasitic states in *Strongyloides stercoralis* and ultimately into the evolution of parasitism in nematodes generally.

## Materials and Methods

### Parasite maintenance and culture

The UPD strain of *Strongyloides stercoralis* was maintained in immuno-suppressed dogs and cultured as described [24]. Free-living adult *S. stercoralis* were isolated from two-day-old coprocultures via Baermann funnels. The worms were washed twice with sterile deionized water to reduce carryover of fecal bacteria and plated on Nematode Growth Medium (NGM agar) plates seeded with *Escherichia coli* OP50. All cultures of *S. stercoralis* were incubated at 22°C unless otherwise noted.

### Transgene construction

#### *gfp* reporters

In *C. elegans*, the promoter for splice form *daf-16b* exists in the large intron between exons 4 and 5 [25]. The *fktf-1* genomic sequence also includes a large, approximately 6 kb, intron between the end of the *fktf-1a*-specific exons (exons 1–3) and the beginning of the *fktf-1b*-specific exon 4 [11]. We used 2.6 kb sequence directly 5′ of the *fktf-1b*-specific ATG codon plus the first 90 bp of the coding sequence as the *fktf-1β* promoter element. Using the primers *Ss*HindBprF1 and *Ss*BprPstR2 (Table S1), we amplified the putative *fktf-1β* promoter from plasmid pPV57. The PCR product was cloned into pAJ01, a promoter-less vector containing the *gfp* coding sequence fused to the *Strongyloides era-1* 3′ UTR, with HindIII and PstI restriction enzymes using standard techniques. The resulting *fktf-1β::gfp::era-1* reporter construct was designated pPV232 (Figure S1).

To create the fusion protein GFP::FKTF-1b, the original construct pPV232 was altered via site-directed mutagenesis (QuikChange Site-Directed Mutagenesis, Stratagene, La Jolla, California, USA), to remove the terminator for the *gfp* coding region and to introduce the unique restriction site BspEI using the primers GFPnoTER-BspEF and R (Table S1). The clone with the appropriate mutation and the new restriction site was designated pPV233 (*fktf-1β::gfp*(no ter)::*era-1*). The cDNA sequence for *fktf-1b* had been previously cloned into a plasmid vector pPV207. The coding region of *fktf-1b* was amplified from pPV207 using the primers: BspE-FkBF (Table S1) and FkB-AvrIIR (Table S1) and inserted into pPV233 using the restriction enzymes BspEI and AvrII via standard techniques. The clone containing the full-length *fktf-1β* promoter with *gfp* fused N-terminal to and in frame with the *fktf-1b* coding region was designated pPV234 (*fktf-1β::gfp::fktf-1b::era-1*) (Figure S1).

#### Phosphorylation mutant constructs

Phosphorylation-null and phosphorylation-mimetic constructs were created from pPV234 using site-directed mutagenesis (QuikChange Site-Directed Mutagenesis, Stratagene, La Jolla, California, USA). All phosphorylation-null mutagenesis reactions were carried out on the cDNA sequence of *fktf-1b* in the pCR-BluntII-TOPO cloning vector (Invitrogen, Carlsbad, California, USA) to improve the efficiency of the reactions. The phosphorylation-null construct was created in three stages, each stage mutating the key regulatory residues in one of the three functional domains. Primers FK-1bPsite1F2 (Table S1) and FK-1bPsite1R (Table S1) mutated threonine 22 to alanine in the N-terminal domain. Primers SsD16Psite2F and SsD16Psite2R (Table S1) mutated both serine 238 and threonine 240 to alanine. Primers FkCPsite3KOF2 and FkCPsite3KOR2 (Table S1) mutated the phosphorylation site serine 317 to alanine in the C-terminal transactivation domain. Once all four sites were mutated, the wild-type *fktf-1b* coding region was removed from the pPV234 vector, and the phospho-null *fktf-1b(4A)* coding region was cloned in using BspEI and AvrII sites using standard techniques. The phospho-null construct was designated pPV243 (*fktf-1β::gfp::fktf-1b(4A)::era*-1) (Figure S1).

Phosphorylation-mimetic primers FkB S238E&T240E F and FkB S238E&T240E R (Table S1) mutated serine 238 and threonine 240, key residues in the forkhead domain, to glutamic acid to mimic the negative charge of phosphorylated residues. The phospho-mimetic construct was designated pPV244 (*fktf-1β::gfp::fktf-1b(S238E/T240E)::era-1*) (Figure S1).

#### Dominant-interfering constructs

The phospho-null construct, GFP::FKTF-1b(4A), which sequesters in the nucleus, still contains functional forkhead (DNA binding) domain and C-terminal transactivation domains and thus should out compete endogenous FKTF-1b for DNA binding sites. Truncation of the C-terminal domain in the phospho-null GFP::FKTF-1b, should create a protein constitutively bound to DNA but unable to activate transcription. Subsequently, replacing the endogenous transactivation domain of FKTF-1b with a repressor domain should create a protein actively repressing transcription specific to FKTF-1b's DNA binding domain.

The transactivation domain was truncated using an introduced ClaI site immediately downstream of the regulatory phosphorylation site S317. The phospho-null *fktf-1b(4A)* in the pCR-BluntII-TOPO vector (Invitrogen, Carlsbad, California, USA) was again used for mutagenesis using the primers FKTFtrcMutCla1F and FkMutTrcCla R2 (Table S1) to introduce the ClaI site in the desired location. The plasmid was cut using BspEI and ClaI to release the *fktf-1b*(4A, truncated) coding region (BspEI-*fktf-1b*(4a, trunc)-ClaI).

The coding region for the *C. elegans* gene *pie-1* was amplified from RNA prepared from a pool of *C. elegans* N2 worms using the primers *Ce-pie-1*ATGF and *Ce-pie-1*StopR (Table S1) and cloned into a pCR-BluntII-TOPO cloning vector (Invitrogen, Carlsbad, California, USA). The active repressor domain of PIE-1 consists of 81 amino acids at the C-terminus (CTD) [21]. Using the primers ClaI-*pieCTD*F and *Ce-pie-1*teravrR (Table S1) the repressor domain alone was PCR amplified using *Pfu*Turbo (Stratagene, La Jolla, California, USA). The resulting PCR product was digested with ClaI and AvrII for cloning, (ClaI-*pieCTD*-AvrII).

pPV234 was digested with BspEI and AvrII to remove the wild-type *fktf-1b* coding region. The ligation reaction included the digested pPV234 vector, the BspEI-*fktf-1b(4A, trunc)*-ClaI and the ClaI-*pie-1CTD*-AvrII simultaneously. The resulting construct, pPV251 (*fktf-1β::gfp::fktf-1b(4A,trunc)::pie-1CTD::era-1*), incorporated *gfp* upstream of the mutated and truncated *fktf-1b* region, which became fused to the *pie-1CTD* (Figure S1). The dominant-negative construct, pPV298 (*fktf-1β::gfp::fktf-1b(4A,trunc)::era-1*) (Figure S1), was created by simply removing the *pie-CTD* from pPV251 via restriction enzyme digest with ClaI and AvrII. The digested ‘sticky ends’ were filled in with *Pfu*Turbo (Stratagene, La Jolla, California, USA) using standard methods and blunt-end ligated.

### Transformation of *S. stercoralis*

Adult female *S. stercoralis* were transformed with transgene encoding plasmids via intra-gonadal microinjection using standard protocols [24],[26]. Coding plasmids were injected at a concentration of 10–100 ng/ml with non-coding plasmids being used as necessary to make up the total DNA concentration to 100 ng/ml. Following injection, worms were transferred to clean NGM OP50 plates with an excess of males and incubated at 22°C.

For general expression patterns, plates were scored at 24 hour intervals for adult survival and frequency of transgene expression among F1 progeny. For specific time points, adults were transferred to clean NGM OP50 plates at three to five hour intervals to obtain egg cohorts. All plates with eggs were checked at hourly intervals for the presence of transgenic progeny. When the time of hatch was known, transgenic larvae were transferred to clean plates marked with the time point and examined after the appropriate interval. Transgenic progeny for which the time of hatch was not known were used for analysis of L3i development. The low transformation rate, <5%, of *S. stercoralis* larvae made it impractical to accumulate sufficient numbers of individuals for both phenotypic study (Table S2 for phenotype counts) and confirmation of a full-length *gfp::fktf-1b* transcript. However, we have confirmed that *C. elegans* transformed with the same *gfp::fktf-1b* coding sequence under the control of the *daf-16* promoter exhibit GFP fluorescence and express a full length transcript encoding the fusion protein (data not shown). Current methods only allow us to observe transgene expression in F1 generation following transformation.

### DIC and Fluorescent Microscopy

Transgenic larvae were identified based on GFP fluorescence using an Olympus SZX12 stereomicroscope with coaxial epifluorescence. For more detailed examination of particular tissues and individual cells, larvae were immobilized on 4% Agar Noble (Sigma, St. Louis, Missouri, USA) pads in 10 mM (L1) or 20 mM (L3i) levamisole and observed using an Olympus BX60 compound microscope equipped with Nomarski Differential Interference Contrast (DIC) optics and epifluorescence (Olympus America Inc., Center Valley, Pennsylvania, USA). Specimens were imaged with a Spot RT Color digital camera and images were processed using either the Spot Advanced image analysis software package (Diagnostic Instruments, Inc., Sterling Heights, Michigan, USA) or Adobe Photoshop 7.0. All image-processing algorithms (e.g. brightness and contrast adjustments) were applied in a linear fashion to the entire image.

### Measurements

Worm lengths were measured using the ImageJ program available from the National Institutes of Health (http://rsb.info.nih.gov/ij/) [27]. Calibrations were done by determining the distance of 10 µm on a micrometer in pixels and then setting the scale in the program. All measurements were done in duplicate using the freehand line option, taking the average of the results for analysis.

### Statistical Analysis

As categorical data, expression patterns, localization and phenotypes, were analyzed using χ^2^ tests. Analysis of the expression patterns of the *fktf-1β* promoter constructs was based on the null hypothesis that the expression patterns were not specific to the hypodermis and the pharynx. Categories of intra-cellular GFP localization were analyzed based on the null hypothesis that the phosphorylation status of the FKTF-1b protein had no effect on its localization. In order to compare the mean lengths of wild-type and transgenic larvae, we used the Mann-Whitney test, which makes no assumptions as to the population distribution of the observations.

## Supporting Information

Figure S1 Diagrams of *fktf-1b* constructs used to transform *S. stercoralis*. pPV232 encoding the *fktf-1β::gfp* transcriptional reporter. pPV234, the GFP::FKTF-1b(wt) fusion protein expression vector. pPV243 (GFP::FKTF-1b(4A)) has all four canonical Akt/PKB phosphorylation sites mutated to alanine. pPV244 (GFP::FKTF-1b(S238E/T240E) has the phosphorylation sites in the forkhead domain changed to the phospho-mimetic glutamic acid. pPV251 (GFP::FKTF-1b(dom-rep)) and pPV298 (GFP::FKTF-1b(dom-neg)) both contain the four alanine mutations. pPV251 encodes a chimeric *fktf-1b* with the repressor domain from Ce-pie-1 replacing the endogenous transactivation domain. pPV298 is truncated just downstream of the fourth regulatory phosphorylation site and thus lacks either a transactivation or a repressor domain.
(0.63 MB TIF)

Figure S2 Transgenic *S. stercoralis* L1 at 1 hour post-hatch. DIC and fluorescence images of *S. stercoralis* larvae. Each DIC image is a separate individual. All scale bars = 10 µm. (A–D) 1-hour-old transgenic hatchlings exhibit similar morphology to the non-transgenic hatchling. (E–G) The fusion protein transgenes have similar levels of expression throughout the larvae. (H–K) Intestinal cells of the 1-hour post-hatch larvae were examined for presence or absence of granules using the primordial gonad (arrow) as a landmark. All larvae, wild-type and transgenic, show healthy looking cells with little granularity at this early timepoint. The similar morphologies of the larvae at 1 hour post-hatch indicate apparently normal embryogenesis of transgenic larvae.
(3.25 MB TIF)

Figure S3 Examples of *Strongyloides stercoralis* wild-type L3i. (A) DIC image of wild-type L3i showing filariform pharynx, pharynx-intestinal junction (black triangle), and constricted, dark intestine. Scale bar = 20 µm. (B) Anterior half of L3i pharynx showing constricted cylindrical structure characteristic of the filariform pharynx. Scale bar = 10 µm. (C) Pharynx-intestinal junction (black triangle) of an L3i. Note the lack of a grinder-like structure at the base of the pharynx and the closed intestine to the left of the junction. Scale bar = 10 µm. Each image is a separate individual.
(3.95 MB TIF)

Table S1 Primer sequences used in construct creation.
(0.06 MB DOC)

Table S2 Wild-type and transgenic larvae examined for developmental abnormalities. Images of larvae at 1 hour, 24 hours, and L3i timepoints were examined for intestinal structure abnormalities, pharyngeal abnormalities, presence of storage granules in intestinal cells, and overall body integrity. Intestinal structure abnormalities were defined as atrophy of the intestine or structural defects. Pharyngeal abnormalities included loss of musculature, terminal bulb irregularities, and loss of synchronized contractions. The larva was positive for granules if the majority of intestinal cells near the primordial gonad contained granules. Overall body damage takes into account loss of cells outside of the intestine, damaged tail architecture, and any structural abnormality other than in the intestine and pharynx.
(1.12 MB TIF)
