# Supplementary figures and images for: Morphogenesis of Strongyloides stercoralis Infective Larvae Requires the DAF-16 Ortholog FKTF-1
Source: PLoS Pathog. 2009 Apr 10;5(4):e1000370. doi: 10.1371/journal.ppat.1000370 (PMC2660150; doi:10.1371/journal.ppat.1000370)

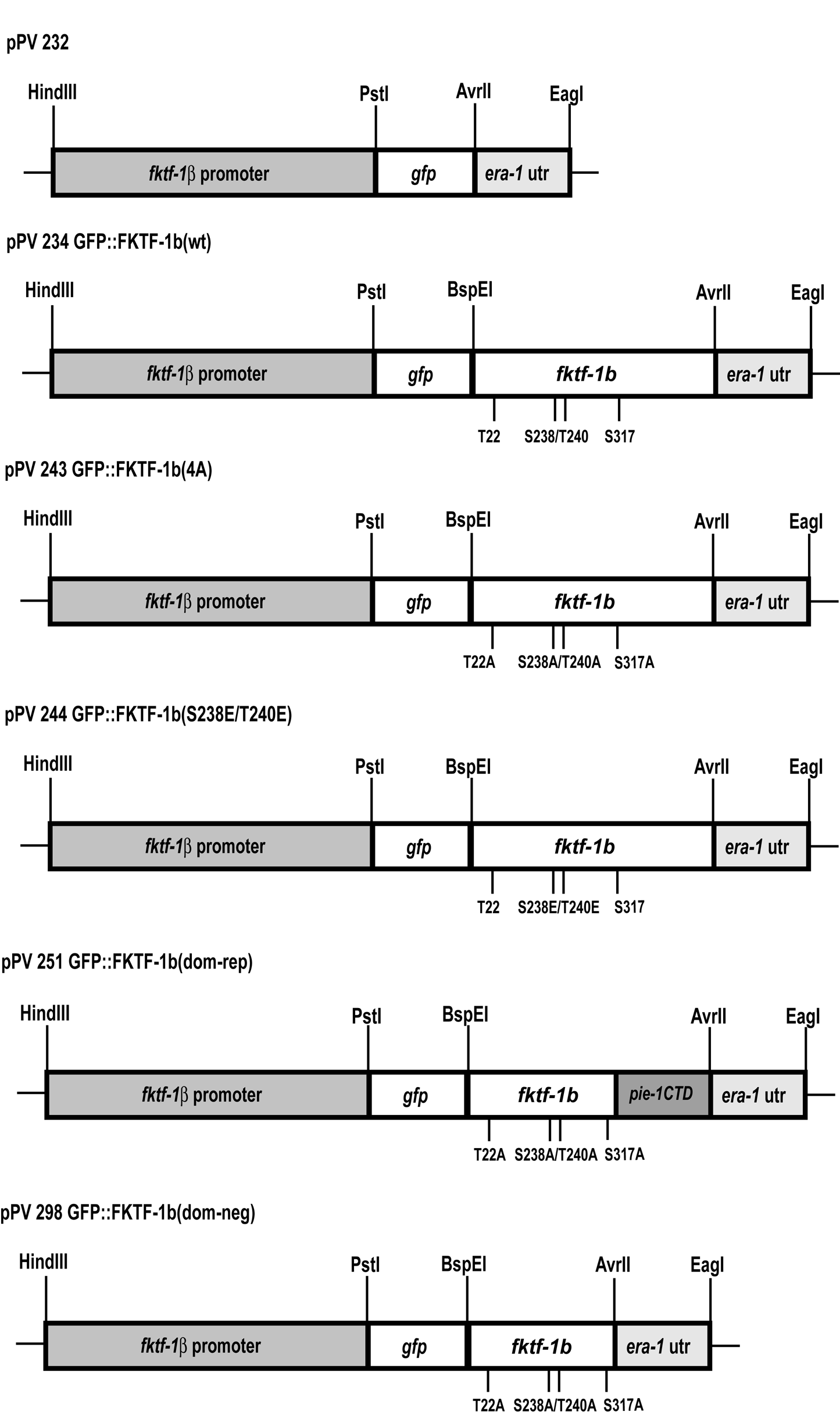

Supplement: Figure S1 — Diagrams of fktf-1b constructs used to transform S. stercoralis. pPV232 encoding the fktf-1β::gfp transcriptional reporter. pPV234, the GFP::FKTF-1b(wt) fusion protein expression vector. pPV243 (GFP::FKTF-1b(4A)) has all four canonical Akt/PKB phosphorylation sites mutated to alanine. pPV244 (GFP::FKTF-1b(S238E/T240E) has the phosphorylation sites in the forkhead domain changed to the phospho-mimetic glutamic acid. pPV251 (GFP::FKTF-1b(dom-rep)) and pPV298 (GFP::FKTF-1b(dom-neg)) both contain the four alanine mutations. pPV251 encodes a chimeric fktf-1b with the repressor domain from Ce-pie-1 replacing the endogenous transactivation domain. pPV298 is truncated just downstream of the fourth regulatory phosphorylation site and thus lacks either a transactivation or a repressor domain. (0.63 MB TIF) [file ppat.1000370.s001.tif]

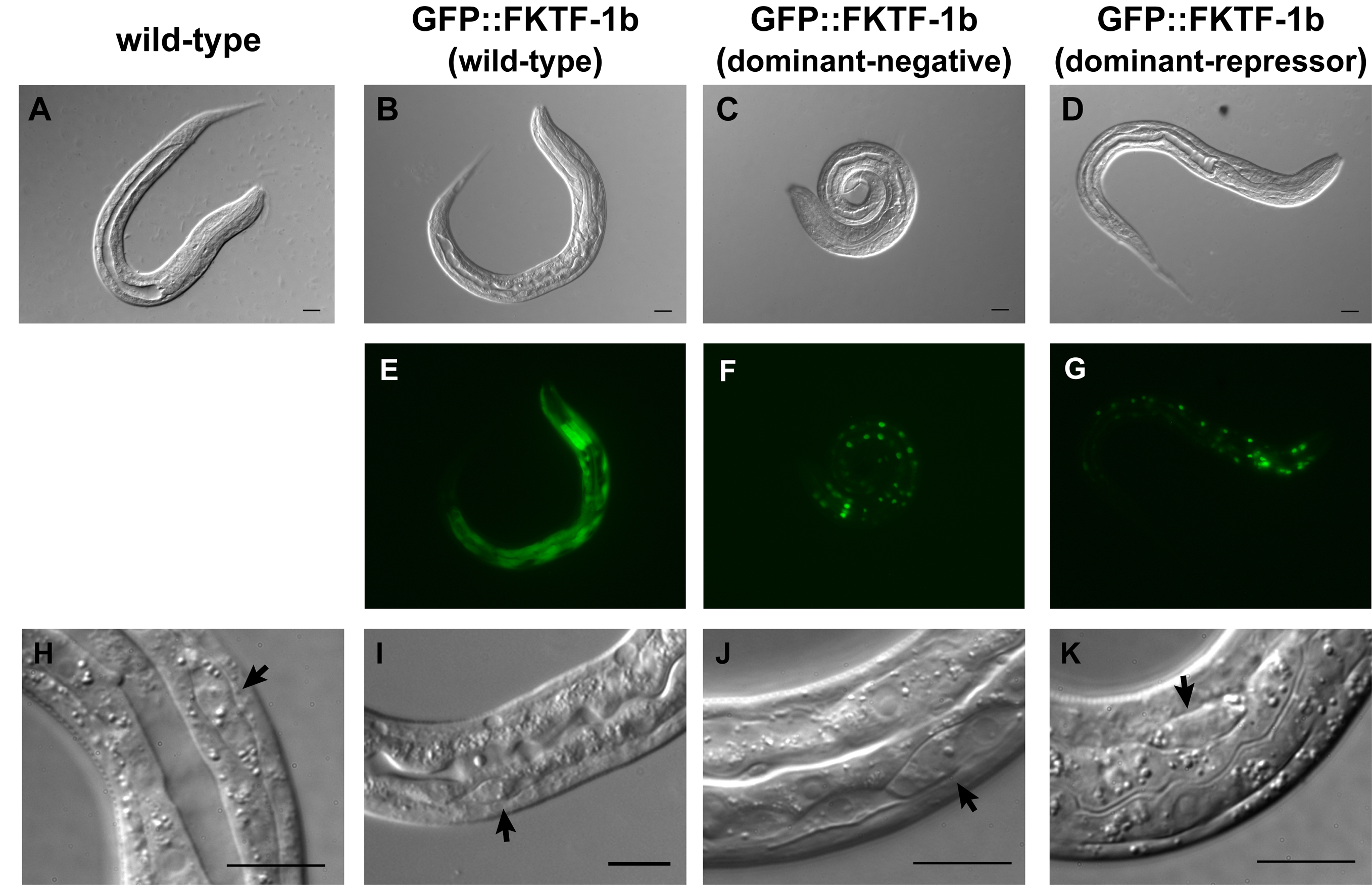

Supplement: Figure S2 — Transgenic S. stercoralis L1 at 1 hour post-hatch. DIC and fluorescence images of S. stercoralis larvae. Each DIC image is a separate individual. All scale bars = 10 µm. (A–D) 1-hour-old transgenic hatchlings exhibit similar morphology to the non-transgenic hatchling. (E–G) The fusion protein transgenes have similar levels of expression throughout the larvae. (H–K) Intestinal cells of the 1-hour post-hatch larvae were examined for presence or absence of granules using the primordial gonad (arrow) as a landmark. All larvae, wild-type and transgenic, show healthy looking cells with little granularity at this early timepoint. The similar morphologies of the larvae at 1 hour post-hatch indicate apparently normal embryogenesis of transgenic larvae. (3.25 MB TIF) [file ppat.1000370.s002.tif]

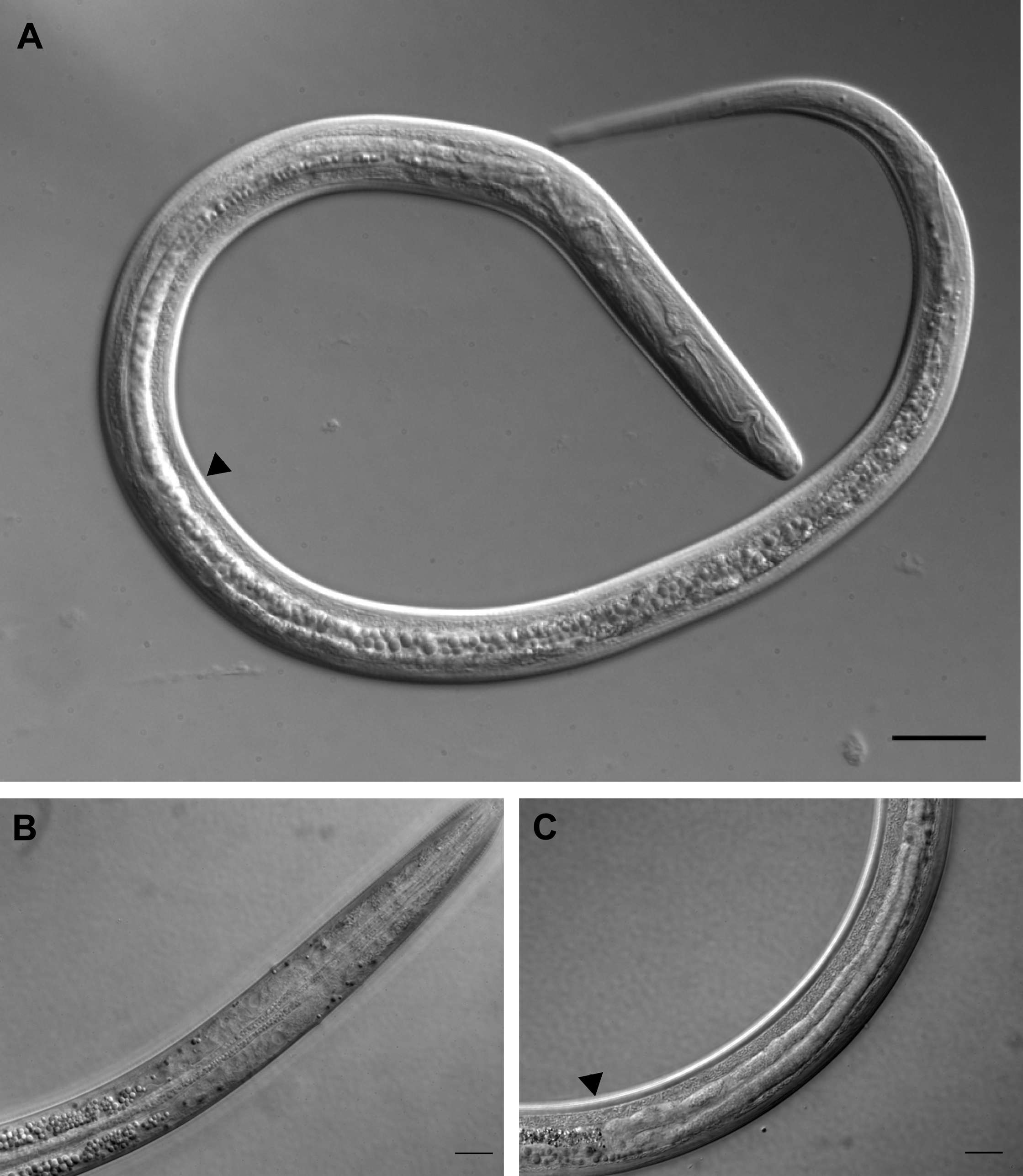

Supplement: Figure S3 — Examples of Strongyloides stercoralis wild-type L3i. (A) DIC image of wild-type L3i showing filariform pharynx, pharynx-intestinal junction (black triangle), and constricted, dark intestine. Scale bar = 20 µm. (B) Anterior half of L3i pharynx showing constricted cylindrical structure characteristic of the filariform pharynx. Scale bar = 10 µm. (C) Pharynx-intestinal junction (black triangle) of an L3i. Note the lack of a grinder-like structure at the base of the pharynx and the closed intestine to the left of the junction. Scale bar = 10 µm. Each image is a separate individual. (3.95 MB TIF) [file ppat.1000370.s003.tif]

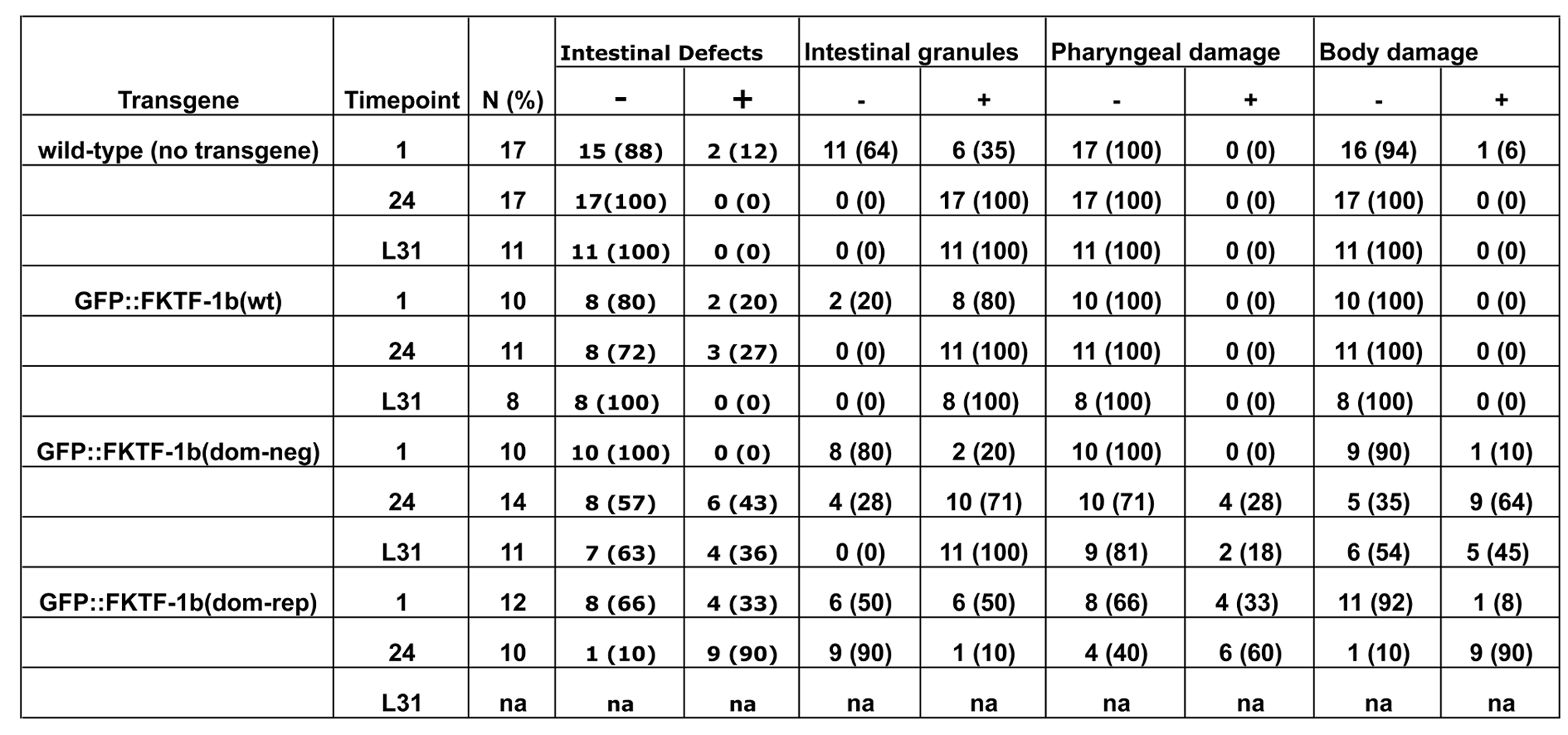

Supplement: Table S2 — Wild-type and transgenic larvae examined for developmental abnormalities. Images of larvae at 1 hour, 24 hours, and L3i timepoints were examined for intestinal structure abnormalities, pharyngeal abnormalities, presence of storage granules in intestinal cells, and overall body integrity. Intestinal structure abnormalities were defined as atrophy of the intestine or structural defects. Pharyngeal abnormalities included loss of musculature, terminal bulb irregularities, and loss of synchronized contractions. The larva was positive for granules if the majority of intestinal cells near the primordial gonad contained granules. Overall body damage takes into account loss of cells outside of the intestine, damaged tail architecture, and any structural abnormality other than in the intestine and pharynx. (1.12 MB TIF) [file ppat.1000370.s005.tif]
