# Supplementary material for: Morphogenesis of Strongyloides stercoralis Infective Larvae Requires the DAF-16 Ortholog FKTF-1
Source: PLoS Pathog. 2009 Apr 10;5(4):e1000370. doi: 10.1371/journal.ppat.1000370 (PMC2660150; doi:10.1371/journal.ppat.1000370)
Supplement: Table S1 — Primer sequences used in construct creation. (0.06 MB DOC) [file ppat.1000370.s004.doc]

| Primer name | Primer sequence (5'-3') |
| --- | --- |
| *Ss*HindBprF1 | 5'-AGTTAAAAGCTTATTATAATGATTGTTG-3' |
| *Ss*BprPstR2 | 5'-AATTATACTGCAGATAGGCCAAGTG-3' |
| GFPnoTER-BspEF | CACATGGCATGGATGAACTATACAATCCGGAATTCGTAGAATTCCTAGG |
| BspE-FkBF | ATCATGTCCGGATGCTGCAAATACATCCATTGTATCGGATGAC |
| FkB-AvrIIR | AAGTCAGACCTAGGCTACAGATCAAAGTTGATTGGAGCATTAC |
| FkB S238E&T240E F &R | GGAAGAAGTCAGCGTAGACAACGTGACAGAGAAAATGAAATTGATACATCTAAATCAGCC |
| FK-1bPsite1F2 | TCCGAGCGCGCTGTTACGCGTGGCCTATGCCACATG |
| FK-1bPsite1R2 | CATGTGGCATAGGCCACGCGTAACAGCGCGCTCGGA |
| SsD16Psite2 F & R | CGTAGACAACGTGACAGAGCTCATGCTATTGATACATCTAAATCAG |
| FkCPsite3KO F2 | GGTTTTCGTCAACGTGCCGCGGCTAATTTAAGTGTCC |
| FkCPsite3KO R2 | GGACACTTAAATTAGCCGCGGCACGTTGACGAAAACC |
| FKTFtrcMutCla1F | GATGCATTTGATGAGATCGATGCTTACCCTTGTTATG |
| FkMutTrcCla R2 | GTAAGCATCGATTTCATCAAATGCATCTAATGTTGGAG |
| *Ce-pie-1*ATGF | ATGGCTCAAACAAAGCCGATTGCCGAGC |
| *Ce-pie-1*StopR | TTAGGAAATAATAGTTGGTGGTGGCTGAGAG |
| *ClaI-pieCTD* F | ATCATGTATCGATGGTCCAAGATGCCGCTTCATTCACG |
| *Ce-pie-1ter* avr R | ATATCCTAGGCAAAATTAGGAAATAATAGTTGGTGGTGGCT |
